# Supplementary material for: Genetic Susceptibility in Head and Neck Squamous Cell Carcinoma in a Spanish Population
Source: Cancers (Basel). 2019 Apr 7;11(4):493. doi: 10.3390/cancers11040493 (PMC6521206; doi:10.3390/cancers11040493)
Supplement: Supplementary file 1 [file cancers-11-00493-s001.pdf]

Supplementary Materials: Genetic susceptibility in head and neck squamous cell carcinoma in a Spanish population

Javier Fernández-Mateos, Raquel Seijas-Tamayo, Juan Carlos Adansa Klain, Miguel Pastor Borgoñón, Elisabeth Pérez-Ruiz, Ricard Mesía, Elvira del Barco, Carmen Salvador Coloma, Antonio Rueda Dominguez, Javier Caballero Daroqui, Encarnación Fernández Ruiz, Alberto Ocana, Rogelio González-Sarmiento and Juan Jesús Cruz-Hernández

Table S1. Primer sequences and genotyping assays in those SNPs defined in Table 5 without specific TaqMan® probes.

SNP	Primer	Primer Sequence (5′-3′)	Type of Assay
MDM2 **rs2279744**	forward	5′-CAGGTCTCCGCGGGAGTTC-3′	Digestion with MspA1I
	reverse	5′-CGTGTCTGAACTTGACCAGC-3′	
KRAS-LC6 **rs61764370**	forward	5′-GCCAGGCTGGTCTCGAA-3′	Custom probe CTCAAGTGAT[T/G]CACCAC
	reverse	5′-CTGAATAAATGAGTTCTGCAAAACAGGTT-3′	
GSTT1	forward	5′-TTCCTTACTGGTCCTCACATCCTC-3′	Null/present
	reverse	5′-TCACCGGATCATGGCCAGCA-3′	
GSTM1	forward	5′CGCCATCTTGTGCTACATTGCCCG-3′	
	reverse	5′-TTCTGGATTGTAGCAGATCA-3′	
β-actin	forward	5′-CCAACCGCGAGAAGATGA-3′	
	reverse	5′-CCCGAGGCGTACAGGGATAG-3′	

Table S2. Distribution between studied SNPs in different genes in laryngeal tumours (Ca) and controls (Co)*.

			**Homozygotes Common Alleles**		**Heterozygotes**				**Homozygotes Rarer Allele**			
**FUNCTION**	**GENE**	**RS**	**Ca**	**Co**	**Ca**	**Co**	***p*-value**	**OR (95% CI)**	**Ca**	**Co**	***p*-Value**	**OR (95% CI)**
**Oncogenes/tumour suppressor genes**	***TP53***	1042522	61	62	54	37	0.165	1.505 (0.846–2.677)	11	27	**0.008**	0.319 (0.136–0.745)
	MDM2	2279744	44	65	57	53	0.279	1.364 (0.778–2.392)	25	11	**0.015**	2.826 (1.219–6.552)
	KRAS-LC6	rs61764370	97	87	27	33	0.382	0.762 (0.414–1.401)	2	6	0.247	0.367 (0.068–1.998)
	EGFR	2227983	78	72	41	44	0.514	0.830 (0.474–1.453)	7	10	0.283	0.536 (0.197–1.608)
**Base excision repair (BER)**	***XRCC1***	25487	58	57	50	55	0.794	0.929 (0.533–1.617)	18	14	0.724	1.160 (0.509–2.646)
		1799782	106	109	20	14	0.371	1.416 (0.663–3.012)	0	2	0.999	0.000 (0.000–)
	APEX	1130409	38	42	65	49	0.308	1.366 (0.750–2.490)	23	35	0.546	0.803 (0.394–1.637)
**Nucleotide excision repair (NER)**	***ERCC2 (XPD)***	13181	72	52	46	58	0.247	0.720 (0.413–1.255)	8	16	**0.046**	0.375 (0.143–0.982)
	ERCC1	11615	53	45	67	58	0.872	0.956 (0.550–1.661)	6	23	**0.013**	0.281 (0.103–0.768)
	XPC	2228000	62	61	57	52	0.530	1.190 (0.692–2.046)	7	13	0.384	0.636 (0.230–1.763)
**Double-strand break repair genes**	***XRCC3***	861539	49	47	59	58	0.721	0.902 (0.512–1.590)	18	21	0.504	0.766 (0.352–1.671)
		1799794	78	78	43	40	0.593	1.166 (0.664–2.047)	5	8	0.789	0.849 (0.255–2.823)
	KU70	2267437	39	44	65	58	0.995	0.998 (0.550–1.811)	22	24	0.780	0.898 (0.422–1.909)
**Inflammatory genes**	***IL1B***	16944	56	58	58	56	0.849	1.054 (0.612–1.817)	12	12	0.930	0.959 (0.382–2.408)
	IL2	2069762	62	67	56	45	0.307	1.333 (0.768–2.311)	8	14	0.132	0.472 (0.178–1.253)
	IL6	1800795	43	62	64	46	**0.003**	2.471 (1.372–4.452)	19	14	0.070	2.164 (0.938–4.991)
	IL10	1800872	75	71	45	47	0.557	0.849 (0.491–1.467)	6	8	0.540	0.694 (0.216–2.229)
	TNFA	361525	104	103	19	21	0.694	0.866 (0.422–1.777)	3	2	0.632	1.565 (0.250–9.780)
**Apoptotic genes**	***NOD2***	2066844	110	109	16	17	0.876	0.940 (0.433–2.043)	0	0	-	-
		2066845	112	119	4	7	0.277	0.485 (0.131–1.789)	0	0	-	-
	BAX	4645878	92	93	31	31	0.806	1.078 (0.592–1.964)	2	2	0.986	1.017 (0.146–7.065)
	BCL2	2279115	37	40	66	55	0.124	1.614 (0.878–2.969)	23	31	0.896	1.050 (0.501–2.203)
**Carcinogen metabolism genes**	***CYP3A5***	rs776746	116	110	8	14	0.246	0.573 (0.223–1.468)	1	1	0.914	1.167 (0.072–19.029)
	GSTP1	1695	65	62	54	51	0.805	1.071 (0.622–1.842)	12	6	0.254	0.535 (0.183–1.568)
	GSTT1	N/A	110	109	15	17	0.483	1.322 (0.606–2.882)				
	GSTM1	N/A	66	59	59	67	0.337	1.289 (0.767–2.168)				
	NFE2L2	13035806	109	95	14	29	**0.019**	0.424 (0.207–0.869)	2	1	0.520	2.235 (0.193–25.903
	(NRF2)	2706110	92	72	24	47	**0.005**	0.425 (0.233–0.775)	9	7	0.732	1.207(0.411–3.541)
	KEAP1	1048290	50	53	58	57	0.768	1.008 (0.622–1.900)	17	16	0.541	1.290 (0.569–2.925)

**p*-values were adjusted by age and related to controls. Statistically significant results are in bold.

Table S3. Distribution between studied SNPs in different genes in tumours from oral cavity (Ca) and controls (Co).

			**Homozygotes Common Alleles**		**Heterozygotes**				**Homozygotes Rarer Allele**			
**FUNCTION**	**GENE**	**RS**	**Ca**	**Co**	**Ca**	**Co**	***p*-value**	**OR (95% CI)**	**Ca**	**Co**	***p*-Value**	**OR (95% CI)**
**Oncogenes/tumour suppressor genes**	***TP53***	1042522	32	38	30	21	0.156	1.696 (0.818–3.518)	8	11	0.779	0.864 (0.310–2.407)
	MDM2	2279744	30	30	28	34	0.593	0.824 (0.404–1.678)	12	6	0.218	2.000 (0.664–6.026)
	KRAS-LC6	rs61764370	40	49	26	18	0.126	1.769 (0.851–3.678)	4	3	0.536	1.633 (0.345–7.727)
	EGFR	2227983	41	35	25	30	0.338	0.711 (0.354–1.428)	4	5	0.591	0.683 (0.170–2.742)
**Base excision repair (BER)**	***XRCC1***	25487	31	31	30	30	1.000	1.000 (0.492–2.034)	9	9	1.000	1.000 (0.350–2.856)
		1799782	62	62	8	8	1.000	1.000 (0.353–2.833)	0	0	-	-
	APEX	1130409	18	25	39	26	0.066	2.083 (0.952–4.559)	13	19	0.914	0.950 (0.375–2.408)
**Nucleotide excision repair (NER)**	***ERCC2 (XPD)***	13181	24	33	36	27	0.101	1.833 (0.888–3.785)	10	10	0.541	1.371 (0.495–3.821)
	ERCC1	11615	19	22	43	36	0.401	1.383 (0.649–2.948)	8	12	0.640	0.772 (0.261–2.284)
	XPC	2228000	36	37	27	26	0.857	1.067 (0.526–2.165)	7	7	0.963	1.028 (0.327–3.226)
**Double-strand break repair genes**	***XRCC3***	861539	27	32	29	28	0.582	1.228 (0.592–2.546)	14	10	0.301	1.659 (0.636–4.332)
		1799794	39	42	26	23	0.587	1.217 (0.598–2.477)	5	5	0.912	1.077 (0.289–4.007)
	KU70	2267437	19	22	35	34	0.657	1.192 (0.550–2.585)	16	14	0.561	1.323 (0.515–3.401)
**Inflammatory genes**	***IL1B***	16944	27	37	36	27	0.093	1.827 (0.904–3.693)	7	6	0.443	1.599 (0.483–5.297)
	IL2	2069762	43	31	22	27	0.152	0.587 (0.284–1.217)	5	12	**0.039**	0.300 (0.096–0.940)
	IL6	1800795	25	39	33	23	**0.031**	2.238 (1.077–4.653)	12	8	0.104	2.340 (0.839–6.528)
	IL10	1800872	29	37	37	29	0.165	1.628 (0.819–3.237)	4	4	0.745	1.276 (0.294–5.542)
	TNFA	361525	61	60	8	9	0.796	0.874 (0.316–2.417)	1	1	0.991	0.984 (0.060–16.088)
**Apoptotic genes**	***NOD2***	2066844	62	63	8	7	0.785	1.161 (0.397–3.397)	0	0	-	-
		2066845	68	67	2	3	0.651	0.657 (0.106–4.057)	0	0	-	-
	BAX	4645878	51	51	19	18	0.888	1.056 (0.497–2.240)	0	1	1.000	0.000 (0.000–)
	BCL2	2279115	13	27	43	30	**0.008**	2.977 (1.325–6.688)	14	13	0.116	2.237 (0.820–6.103)
**Carcinogen metabolism genes**	***CYP3A5***	rs776746	61	63	9	6	0.432	1.549 (0.520–4.614)	0	0	-	-
	GSTP1	1695	38	33	29	28	0.766	0.899 (0.448–1.808)	3	8	0.118	0.326 (0.080–1.329)
	GSTT1	N/A	8	11	62	59	0.461	1.445 (0.543–3.842)				
	GSTM1	N/A	42	34	28	36	0.176	0.630 (0.322–1.230)				
	NFE2L2 (NRF2)	13035806	56	54	12	16	0.448	0.723 (0.313–1.670)	2	0	0.999	-
		2706110	45	39	22	29	0.241	0.657 (0.326–1.325)	3	2	0.780	1.300 (0.206–8.184)
	KEAP1	1048290	24	27	39	36	0.586	1.219 (0.598–2.485)	7	7	0.845	1.125 (0.345–3.673)

p-values are related to controls. Statistically significant results are in bold.

Table S4. Distribution between studied SNPs in different genes in pharyngeal tumours (Ca) and controls (Co).

			Homozygotes Common Alleles		Heterozygotes				Homozygotes Rarer Allele			
**FUNCTION**	**GENE**	**RS**	**Ca**	**Co**	**Ca**	**Co**	***p*-value**	**OR (95% CI)**	**Ca**	**Co**	***p*-Value**	**OR (95% CI)**
**Oncogenes/tumour suppressor genes**	***TP53***	1042522	53	47	44	33	0.583	1.182 (0.650–2.151)	3	20	**0.002**	0.133 (0.037–0.476)
	MDM2	2279744	49	49	37	42	0.675	0.881 (0.487–1.595)	14	9	0.350	1.556 (0.616–3.928)
	KRAS-LC6	rs61764370	68	69	30	27	0.704	1.127 (0.607–2.093)	2	4	0.442	0.507 (0.090–2.862)
	EGFR	2227983	58	54	36	37	0.743	0.906 (0.502–1.634)	6	9	0.394	0.621 (0.207–1.860)
**Base excision repair (BER)**	***XRCC1***	25487	40	44	47	44	0.594	1.175 (0.649–2.127)	13	12	0.701	1.192 (0.487–2.913)
		1799782	90	87	10	12	0.634	0.806 (0.3311.961)	0	1	1.000	0.000 (0.000–)
	APEX	1130409	29	35	45	39	0.320	1.393 (0.725–2.675)	26	26	0.615	1.207 (0.580–2.513)
**Nucleotide excision repair (NER)**	***ERCC2 (XPD)***	13181	48	49	36	40	0.782	0.919 (0.504–1.676)	16	11	0.370	1.485 (0.625–3.526)
	ERCC1	11615	39	36	48	51	0.646	0.869 (0.477–1.584)	13	13	0.860	0.923 (0.378–2.253)
	XPC	2228000	48	54	46	38	0.296	1.362 (0.763–2.431)	6	8	0.768	0.844 (0.273–2.606)
**Double-strand break repair genes**	***XRCC3***	861539	36	35	43	49	0.616	0.853 (0.459–1.586)	21	16	0.550	1.276 (0.574–2.839)
		1799794	56	59	36	38	0.722	1.112 (0.620–1.995)	5	6	0.711	1.264 (0.365–4.377)
	KU70	2267437	39	33	47	49	0.504	0.812 (0.440–1.497)	14	18	0.328	0.658 (0.285–1.522)
**Inflammatory genes**	***IL1B***	16944	47	52	43	39	0.506	1.220 (0.679–2.192)	10	9	0.681	1.229 (0.460–3.286)
	IL2	2069762	46	52	41	37	0.459	1.253 (0.690–2.273)	13	11	0.526	1.336 (0.546–3.272)
	IL6	1800795	46	48	45	35	0.336	1.342 (0.737–2.442)	9	13	0.498	0.722 (0.282–1.852)
	IL10	1800872	60	57	37	38	0.792	0.925 (0.518–1.652)	3	5	0.456	0.570 (0.130–2.495)
	TNFA	361525	83	83	17	15	0.746	1.133 (0.531–2.419)	0	2	0.999	0.000 (0.000–)
**Apoptotic genes**	***NOD2***	2066844	82	86	17	14	0.538	1.274 (0.590–2.749)	1	0	1.000	1694278518 (000–)
		2066845	96	95	4	5	0.734	0.792 (0.206–3.039)	0	0	-	-
	BAX	4645878	83	74	15	23	0.141	0.581 (0.282–1.197)	2	3	0.575	0.594 (0.097–3.655)
	BCL2	2279115	30	33	46	42	0.573	1.205 (0.630–2.302)	24	25	0.886	1.056 (0.500–2.229)
**Carcinogen metabolism genes**	***CYP3A5***	rs776746	89	89	10	10	1.000	1.000 (0.397–2.520)	0	0	-	-
	GSTP1	1695	49	40	41	46	0.293	0.728 (0.402–1.317)	7	12	0.155	0.476 (0.171–1.322)
	GSTT1	N/A	84	86	16	14	0.692	0.855 (0.393–1.860)				
	GSTM1	N/A	46	49	54	51	0.671	0.887 (0.509–1.545)				
	NFE2L2	13035806	78	75	18	25	0.292	0.692 (0.349–1.372)	3	0	0.999	-
	(NRF2)	2706110	68	54	25	41	**0.020**	0.484 (2.262–0.893)	7	5	0.863	1.112 (0.334–3.698)
	KEAP1	1048290	42	44	49	46	0.713	1.116 (0.623–2.000)	9	10	0.908	0.943 (0.349–2.550)

p-values are related to controls. Statistically significant results are in bold.
